# Prevalence of Extra Roots in Permanent Mandibular First Molars in Iranian Population: A CBCT Analysis

**DOI:** 10.22037/iej.2017.14

**Published:** 2017

**Authors:** Saeed Rahimi, Hadi Mokhtari, Bahram Ranjkesh, Masoomeh Johari, Mohammad Frough Reyhani, Shahriar Shahi, Sina Seif Reyhani

**Affiliations:** a *Dental and Periodontal Research Center, Department of Endodontics, Dental School, Tabriz University of Medical Sciences, Tabriz, Iran; *; b * Department of Dentistry and Oral Health, Aarhus University, Health, Denmark Section of Dental Pathology, Operative Dentistry and Endodontics, Vannelyst boulevard, Aarhus, Denmark****; ***; c *Department of Oral and maxillofacial Radiology, Dental School, Tabriz University of Medical Sciences, Tabriz, Iran; *; d *General Practitioner, Dental and Periodontal Research Center, Dental School, Tabriz University of Medical Sciences, Tabriz, Iran*

**Keywords:** Cone-beam Computed Tomography, Molar, Prevalence, Root

## Abstract

**Introduction:**

Having knowledge about the anatomy of root canal system is essential for success of endodontic treatment. The present study used cone-beam computed tomography (CBCT), to evaluate the prevalence of third root in mandibular first molars in a selected Iranian population.

**Methods and Materials:**

A total of 386 CBCT images from subjects referred to oral and maxillofacial radiology department of dental faculty of Tabriz University of Medical Sciences from 2011 to 2013 were selected and evaluated for this study and the cases with well-developed permanent mandibular first molars were included. The 3D images were reconstructed in axial cross sections and evaluated by two endodontists for the presence of the third extra lingual (radix entomolaris) or buccal (radix paramolaris) root. The chi-squared test was used to evaluate the relationship between gender and bilateral incidence of extra roots in mandibular first molars.

**Results:**

The distribution of three-rooted mandibular first molars with an additional root was 3%, (3.53% in female and 2.50% in male patients). There was no significant relationship between gender and bilateral occurrence of three-rooted mandibular first molars.

**Conclusion:**

The occurrence of three-rooted mandibular first molars in Iranian population is not uncommon which should be taken into consideration by the dental practitioners during root canal treatment of these teeth.

## Introduction

Prognosis of the endodontic treatment is affected by knowledge about the anatomy and morphology of the root canal system due to wide variations in tooth anatomy in different racial and ethnic groups [1]. The mandibular first molar commonly has mesial and distal roots. Both roots are wide buccolingually almost similar in length or the mesial root is a little longer [2].

The mandibular first molar might exhibit variations in the number of roots, such as an extra distal root [2]. The third root in mandibular three-rooted molars is found in two forms: the supernumerary root located distolingually which is called radix entomolaris or an additional root at the mesiobuccal side which is called radix paramolaris [3]. Clinically, awareness about the presence of an extra distolingual or mesiobuccal root in mandibular molars is the key to the success of root canal treatment [3]. 

Conventional and digital radiographic techniques are not highly accurate in determining the root morphology because they yield a two-dimensional image of a three-dimensional object [4]. In addition, studies carried out on extracted teeth cannot be conclusive in relation to gender and the prevalence of bilateral three-rooted mandibular first molars [5]. A new technique for the evaluation of the number of roots and root canals is cone-beam computed tomography (CBCT) which has made three-dimensional reconstruction of the root canal system possible, so that the dentist is able to view an accurate and real image of the root canal system [4-6]. 

The prevalence of these three-rooted mandibular first molars appears to be less than 3% in African populations, less than 5% in Eurasian and Asian populations, and is reported to be higher than 5% (even up to 40%) in Mongolian traits [7].

Given racial and ethnic variations in the anatomy and morphology of human teeth and since to date no studies have evaluated the frequency of radix entomolaris and radix paramolaris in three-rooted mandibular first molars using CBCT images in Iran, the present study was designed to evaluate the prevalence of this anatomical variation in a selected Iranian population.

## Materials and Methods

**Figure 1:**
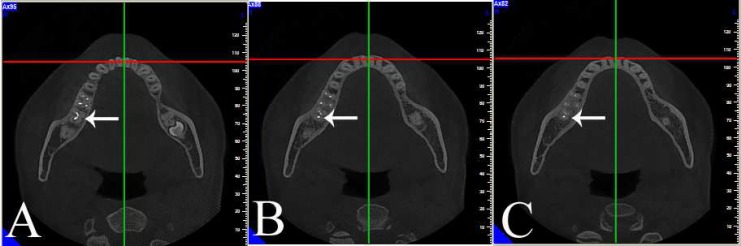
A case of a unilateral permanent three-rooted right mandibular first molar in axial direction. *A)* Coronal-third root section; *B)* Mid-root section; *C)* Apical-third root section

CBCT images of 386 patients who had referred to the Department of Oral and Maxillofacial Radiology, Tabriz Faculty of Dentistry, as a part of the examination, diagnosis and treatment planning procedures, were collected. Informed consent forms were obtained from all the patients whose CBCT images were evaluated for the study. Inclusion criteria included the complete maturation of the apex of mandibular first molars. All the CBCT images were taken with NewTom VG 9000 CBCT device (Quantitative Radiology SRL Co., Verona, Italy) with maximum kVp of 120 and 150 mA. The x-ray specifications for the images were kVp=10 and mA=0.5‒10. The 3D axial cross-sections (coronal, middle, and apical-third root section) had been prepared with the NTT Viewer software program, version 2.21 (NTT Software Corporation, Yokohama, Japan) (Figure 1). In order to evaluate mandibular first molars with three roots with radix entomolaris and radix paramolaris, extra roots of all the images were double checked by two endodontists. All the cases on which the two endodontists did not reach an agreement were excluded. Data (including frequencies and percentages) were evaluated using descriptive statistics. 

Chi-squared test was used to evaluate the relationship between gender and bilateral incidence of extra roots in mandibular first molars. Statistical significance level was set at 0.05.

## Results

A total of 386 CBCT images from 226 female and 160 male patients were included in the present study. Twelve mandibular first molars had extra distal roots (3.10%). There were 8 (3.53%) cases of three-rooted mandibular first molars in female patients while its prevalence rate in men was 4 (2.50%), with no significant differences between genders (*P*=0.06). All the extra roots were radix entomolaris. 

The frequency of bilateral three-rooted mandibular first molars was 1.03%, with unilateral frequencies of 0.77% and 1.29% on the left and right sides, respectively (Table 1). The Chi-squared test revealed no significant relationship between gender and the bilateral prevalence of three-rooted mandibular first molars (*P*=0.22). 

## Discussion

Based on the results of clinical studies, awareness about the presence of a distolingual root in mandibular first molars is the key to the successful endodontic treatment and periodontal care, especially in complicated cases [8, 9]. Distolingual extra roots of mandibular first molars are usually smaller than the mesiobuccal extra roots and exhibit buccolingual curvature. Therefore, proper endodontic treatment of these teeth is of great importance [7, 10]. 

CBCT is an accurate, non-invasive and practical technique for a reliable comparison of the results in relation to gender and bilateral incidence of three-rooted mandibular first molars between different populations. In addition, CBCT images show the real three-dimensional structure of these teeth, making it possible to make an accurate estimation of distances and angles [4, 11, 12]. Therefore, CBCT is considered a proper tool in endodontic treatment or retreatment of teeth with extra roots. Some previous studies have used extracted teeth to determine the frequency of mandibular permanent first molars with three roots, possibly resulting in underestimation of their frequency because teeth with slender roots might easily fracture during tooth extraction [13-19]. Therefore, use of CBCT in the present study made it possible to more accurately and reliably evaluate mandibular first molars without destroying them, compared to other techniques. 

**Table 1.** Numbers (percentages) of patients with permanent three-rooted mandibular first molars

**Patient (N)**	**Unilateral**		**Bilateral N (%)**	**Total N (%)**
	**Right N (%) **	**Left N (%) **		
**Male (160)**	2 (1.25)	1 (0.62)	1 (0.62)	4 (2.50)
**Female (226)**	3 (1.32)	2 (0.88)	3 (1.32)	8 (3.53)
**Total (386)**	5 (1.29)	3 (0.77)	4 (1.03)	12 (3.10)

The results of the present study showed a frequency rate of 3.10% for mandibular permanent first molars with an extra distolingual root, which is much less than the frequency reported by Tu *et al.* (33.3%) [3] and Zhang *et al*. (29%) [20] with the use of CBCT technique in Taiwanese population. The differences in the frequencies of these studies and the present study might be attributed to racial and ethnic differences. The results of studies carried out by Huang *et al*. [8] and Tu *et al.* [21] using periapical radiographies, showed lower frequencies (26.9% and 21.1%, respectively) compared to the two studies above, which might be attributed to more accurate analysis provided by CBCT technique, resulting in more accurate diagnosis. 

A study by Schäfer *et al*. [22] on a German population using the periapical radiographic technique, in which 1024 periapical radiographies were collected from 800 patients, showed a prevalence of 1.35% for distal extra roots in mandibular first molars and all the three-rooted teeth exhibited unilateral incidence. Another study by Colak *et al*. [23] on a Turkish population, using periapical radiographic technique, showed a prevalence rate of 1.41%. In addition, a study by Demirbuga *et al.* [24] on a Turkish population, using the CBCT technique, showed a prevalence rate of 2.06% for three-rooted mandibular first molars, which is very close to the frequency rate in the present study. In general, such differences in frequency rates reflect ethnicity-related variations, sample size and case selection.

The results of the present study did not reveal any significant differences in the prevalence of three-rooted mandibular first molars between males and females, which is consistent with the result of the other studies by Tu *et al*. [3, 21], using periapical radiographies and CBCT techniques, respectively. 

No significant differences were observed in the prevalence of three-rooted mandibular first molars between the right and left sides. However, based on the results of a study by Tu *et al.* [3] on a Taiwanese population and some other studies carried out on Hispanic children and Chinese populations [3, 17, 25, 26], with periapical technique or analysis of extracted teeth, more three-rooted teeth were detected on the right side. However, a study on a Singaporean population showed a higher prevalence rate of mandibular first molars with extra distolingual roots on the left side [19].

The bilateral prevalence of three-rooted mandibular first molars in the present study was significantly less than studies done by Tu *et al.* [3, 21], Walker *et al. *[18] and de Souza-Freitas *et al. *[27] on Asian populations. The discrepancies between the results of the present study and other studies might be attributed to racial, ethnic and method differences.

## Conclusion

In some cases of Iranian population, the permanent mandibular molars (especially first mandibular molar) have 3 roots, which should be taken into account by the dental practitioners during root canal treatment.
